# Dietary Supplementation With Didancao (*Elephantopus scaber* L.) Improves Meat Quality and Intestinal Development in Jiaji Ducks

**DOI:** 10.3389/fvets.2021.753546

**Published:** 2021-10-13

**Authors:** Chengjun Hu, Lihong Gu, Mao Li, Fengjie Ji, Weiping Sun, Dingfa Wang, Weiqi Peng, Dajie Lin, Quanwei Liu, Haofu Dai, Hanlin Zhou, Tieshan Xu

**Affiliations:** ^1^Tropical Crops Genetic Resources Research Institute, Chinese Academy of Tropical Agricultural Sciences, Haikou, China; ^2^Institute of Animal Science and Veterinary Medicine, Hainan Academy of Agricultural Sciences, Haikou, China; ^3^Institute of Tropical Bioscience and Biotechnology, Chinese Academy of Tropical Agricultural Sciences, Haikou, China

**Keywords:** *Elephantopus scaber* L., ducks, feed conversion ratio, growth performance, meat quality

## Abstract

Didancao (*Elephantopus scaber* L.) has been used as a traditional herbal medicine and has exhibited a beneficial role in animal health. This study aimed to investigate the effects of dietary supplementation with *E. scaber* on growth performance, meat quality, intestinal morphology, and microbiota composition in ducks. A total of 480 Jiaji ducks (42 days old, male:female ratio = 1:1) were randomly assigned to one of four treatments. There were six replicates per treatment, with 20 ducks per replicate. The ducks in the control group (Con) were fed a basal diet; the three experimental groups were fed a basal diet supplementation with 30 (T1), 80 (T2), and 130 mg/kg (T3) of *E. scaber*. After a 48-day period of supplementation, growth performance, meat quality, intestinal morphology, and microbiota composition were evaluated. The results showed that no differences were observed in the final body weight, average daily feed intake, and average daily gain among the four groups. Compared with that in the Con group, the feed conversion in the T1 and T2 groups was increased significantly; the T2 group was shown to decrease the concentration of alanine aminotransferase in serum; the T3 group was lower than the Con group in the concentration of aspartate aminotransferase and was higher than the Con group in the concentration of high-density lipoprotein-cholesterol. The highest concentration of creatinine was observed in the T1 group. The T2 group was higher than the Con group in the contents of Phe, Ala, Gly, Glu, Arg, Lys, Tyr, Leu, Ser, Thr, Asp, and total amino acids in the breast muscle. Moreover, the T2 group was higher than the Con group in the contents of meat C18:2*n*−6 and polyunsaturated fatty acid. The concentration of inosinic acid in the T1, T2, and T3 groups was significantly higher than that in the Con group. However, the Con group was higher than the T2 or T3 group in the Zn content. The T2 group was lower than the Con group in the jejunal crypt depth. The T3 group was higher than the Con group in the ileal villus height and the ratio of villus height to crypt depth. In addition, the T3 group had a trend to significantly increase the abundance of Fusobacteria. Compared with the Con group, the T1 and T2 groups displayed a higher abundance of *Subdoligranulum*. Collectively, dietary supplementation with 80 mg/kg of *E. scaber* improves meat quality and intestinal development in ducks.

## Introduction

Antibiotics have been widely used as growth promoters in animal feed. However, the use of antibiotics leads to the development of antibiotic resistance, which can spread between animals and humans (1). To reduce the negative effects caused by the abuse of antibiotics and improve the safety of meat quality, the use of antibiotics as growth promoters was banned by the European Union, the United States, Korea, and China. Without the use of antibiotics in animal feed, animals might suffer from decreased growth performance and increased enteric disease (2). Therefore, suitable antibiotic alternatives are needed to enhance the growth and health status of animal.

Natural products especially the plant-based extracts were reported to exert an important role in animal health. For instance, dietary supplementation with plant extracts improved feed efficiency of pigs challenged with respiratory syndrome virus (3); and supplementation of 20% *Withania somnifera* root extract caused a reduction in the mortality and enhanced the cellular immune responses in *Escherichia coli*-infected broilers (4). In addition, extract from *Eucommia ulmoides* or eucalyptus leaf was shown to improve oxidative stability and meat quality of breast meat in broilers (5, 6). *Elephantopus scaber* L. (*E. scaber*) belongs to Asteraceae family and has been used as herbal medicine for humans (7). Available evidences showed that *E. scaber* presents a multitude of health benefits including anti-diarrheal (7), anti-inflammatory (8), and anti-bacterial activities (9), suggesting that dietary supplementation with *E. scaber* may have the potential to improve animal growth and health.

Our previous study showed that compounds extracted from *E. scaber* exhibited antibacterial activity (10), indicating that *E. scaber* could serve as an antibiotic alternative to antibiotic growth promoters in poultry production. However, to date, there have few investigations on the application of *E. scaber* in duck production. Therefore, we investigated the effects of dietary supplementation with *E. scaber* on growth performance, meat quality, intestine development, and microbiota composition in ducks.

## Materials and Methods

### Animals and Experimental Treatments

A total of 480 Jiaji ducks (42 days old, male:female ratio = 1:1) with similar body weight were randomly assigned to one of four treatment. There were six replicates per treatment, with 20 ducks per replicate. The leaves of *E. scaber* were obtained from a local farm, and dried, and then ground into power. The ducks in the control group (Con) were fed a basal diet; the three experimental groups were fed a basal diet supplementation with 30 (T1), 80 (T2), and 130 mg/kg (T3) of *E. scaber*. The experiment was conducted from 42 to 90 days of age. The basal diet (Table 1) was formulated according to the recommendation of the Nutrient Requirements of Poultry (National Research Council, 1994). All ducks were reared in raised plastic wire-floor pens with a pen size of 20 m^2^ (4 m × 5 m) and had free access to water and feed. The duck house (10 m × 80 m) was equipped with a window. Each pen was provided with a 20-W incandescent white fluorescent lamp, and 24-h continuous incandescent lighting was provided. The temperature was set initially at 30°C and reduced gradually to 20°C at the end of the experiment. The relative humidity in duck house was 75–65%. The feed intake was recorded daily, and the final body weight was recorded at 90 days age. Average daily gain (ADG), average daily feed intake (ADFI), and the ratio of feed intake to body weight gain (F:G) of ducks for each replicate were measured. This study was performed in accordance with the recommendations of the Guide for the Care and Use of Chinese Academy of Tropical Agricultural Sciences.

**Table 1:** Composition and nutrition levels of the basal diet.

**Ingredient**	**Content (%)**
Corn	70.53
Soybean meal	23.50
Wheat bran	1.70
CaHPO_4_	1.37
Limestone	0.90
l-Lysine	0.14
dl-Methionine	0.18
Premix^a^	1.68
Total	100
**Nutrient levels**	
ME (MJ/kg)	12.27
Crude protein	16.79
Crude fat	3.05
Crude fiber	2.63
Calcium	0.46
Available P	0.35
l-Lysine	0.95
Methionine	0.45

*ME, metabolizable energy*.

a *One kilogram of multiple vitamin premix contained: vitamin A, 50,000,000 IU; vitamin B_1_, 10,000 mg; vitamin B_2_, 20,000 mg; vitamin B_6_, 10,000 mg; vitamin B_12_, 5,000 mg; vitamin C, 4,000 mg; vitamin D, 1,000,000 IU; vitamin E, 60,000 IU; vitamin K_3_, 8,000 mg; folic acid, 2,500 mg; niacin, 80,000 mg; pantothenic acid, 30,000 mg; biotin, 2,200 mg; Cu, 5 g; Fe, 50 g; Zn, 55 g; Mn, 55 g; I, 0.3 g; Se, 0.22 g*.

### Sample Collection

After 12 h of feed deprivation, one duck with a similar average body weight was selected from each replicate for sample collection. The animals were stunned electrically, killed immediately by neck cutting, and eviscerated manually. Blood samples were collected from the wing vein using 10-ml centrifuge tubes and centrifuged at 3,000 × g and 4°C for 15 min to recover the serum. The skin with subcutaneous fat, liver, heat, gizzard, abdominal fat, breast meat (pectoralis muscle), and leg meat (all thigh muscle) was removed from the right side of carcasses and weighed; and the percentages relative to live body weight, eviscerated, and semi-eviscerated were calculated. Breast muscle samples were collected from the breast and then stored at −80°C for further analysis. The anterior jejunum and posterior ileum segments (~2 cm in length) were fixed in 4% paraformaldehyde for analysis of intestinal morphology. The colon was separated, and the fecal sample was collected into a 10-ml sterile tube and stored at −80°C for further analysis.

### Biochemical Parameter

The concentrations of albumin (ALB), alanine aminotransferase (ALT), alkaline phosphatase (ALP), aspartate aminotransferase (AST), creatinine (CRE), glucose (GLU), high-density lipoprotein-cholesterol (HDL-C), low-density lipoprotein-cholesterol (LDL-C), total cholesterol (T-CHO), triglyceride (TG), total protein (TP), and uric acid (UA) in the serum were measured using the biochemical analytical instrument PUZS-600B (Medical Equipment Co., Ltd., Beijing, China) and respective commercial assay kits.

### Concentration of Inosinic Acid in Muscle

The concentration of inosinic acid (IMP) in the breast muscle was determined according to the procedure described previously (11). In brief, breast muscle samples were dried in a vacuum freeze dryer and ground into powder. Muscle powder (about 0.1 g) was mixed with 10 ml of perchloric acid (5%), and then the mixture was homogenized. The homogenate was centrifuged at 3,000 rpm for 10 min at 4°C to obtain the solution. The solution was filtered through a 0.45-μm membrane filter; then the samples were analyzed using high-performance liquid chromatography system (Agilent 1200 series, Waldbronn, Germany).

### Trace Element Concentration in Muscle

The concentrations of Fe, Se, and Zn in breast muscle sample were determined according to the method described previously (12). Muscle sample (0.5 g) was mixed with 5 ml of acid solution (HNO_3_:HClO_4_ = 4:1) in 50-ml conical flask for 24 h, and then the lysate was heated to 180°C on an electric heating plate. Ten milliliter of 5 M hydrochloric acid solution was added to the conical flask after the lysate returned to room temperature. The lysate was then heated until clarification. Then, the solution was diluted to a volume of 25 ml with 2 M of hydrochloric acid and 10% potassium ferricyanide solution. Fe, Se, and Zn standard solutions were used to construct standard curves. The concentrations of Fe, Se, and Zn were detected using an atomic fluorescence spectrometer (AFS-9330; Beijing Titan Instruments Co., Ltd., Beijing, China).

### Amino Acid Composition Analysis

Muscle powder (about 0.1 g) was hydrolyzed in 5 ml of 6 M hydrochloric acid solution at 110°C for 24 h. The suspension was diluted with water, 1 ml of the supernatant was filtered through a 0.45-μm membrane, and then the samples were analyzed using an amino acid (AA) analyzer (L8800, Hitachi, Tokyo, Japan).

### Fatty Acid Contents Analysis

Fatty acid contents in the breast muscle were analyzed by gas chromatography (GC). In detail, total lipids were extracted from the breast muscles using the chloroform–methanol method. The lipids were converted to fatty acid methyl esters using KOH/methanol and analyzed using GC. The GC conditions were conducted according to our previous publication (13). Each fatty acid peak was identified by comparing their retention times with standards. The fatty acid composition was calculated as a percentage of the total fatty acids.

### Intestinal Histomorphological Analysis

Hematoxylin and eosin (H&E) staining was used to analyze intestinal morphology. Intestine tissues fixed in 4% paraformaldehyde were paraffin-embedded, sectioned at 5-μm thickness, and stained with H&E. The images were captured using a light microscope (Olympus Bx51, Tokyo, Japan). The villus height and crypt depth were measured using ImageJ software.

### DNA Extraction and PCR Amplification

DNA samples were extracted from feces through the HiPure Stool DNA kit B (Magen, Shanghai, China). The quality and concentration of DNA were measured using a NanoDrop® ND-1000 instrument (NanoDrop Technologies Inc., Wilmington, DE, USA). The V3–V4 region of the 16S rRNA gene was amplified using primers 515F (5′-GTGCCAGCMGCCGCGGTAA-3′) and 806R (5′-GGACTACHVGGGTWTCTAAT-3′) (14). The amplified PCR products were identified, purified, and quantified. Equal amounts of purified products were subject to paired-end sequencing on the Illumina MiSeq platform (Illumina, Inc., San Diego, CA, USA).

### Analyses of Microbial Communities

QIIME and FLASH (v.1.2.11) software package were used to analyze the Raw Illumina fastq files; then the raw tags were analyzed using Trimmomatic (v.0.30) and FLASH (v.1.2.11) software packages to obtain high-quality clean reads. The criteria for high-quality sequence selection was described in our previous study (14). The high-quality sequences were clustered into operational taxonomic units (OTUs) with sequence similarity of 97% using USEARCH (v7.0.1090). OTU sequences were taxonomically classified according to the Ribosomal Database Project (RDP) Classifier based on the Greengene (V201305) reference database.

### Statistical Analysis

Data were statistically analyzed by one-way ANOVA using SPSS 20.0 software (SPPS Inc., Chicago, IL, USA). Homogeneity of variances was tested using Levene's test. The differences among the groups were analyzed using Duncan's multiple range tests or Tamhane's T2 tests. The relative species abundances (at phylum and genus levels) of gut microbial communities were analyzed using the Kruskal–Wallis test. Pearson's correlation coefficient was conducted to assess the relationships among environmental factors. Differences were considered statistically at *p*-value of <0.05. Data were expressed as the mean ± SEM.

## Results

### Growth Performance

As shown in Table 2, no differences (*p* > 0.05) were observed in the final body weight, ADG, or ADFI among the four groups. Compared with the Con group, the ratio of F:G in the T1 group was decreased (*p* < 0.05) by 9.3%; however, no difference (*p* > 0.05) was observed in the F:G among the Con, T2, and T3 groups.

**Table 2:** Effects of dietary supplementation with *Elephantopus scaber* on growth performance of ducks.

**Item**	**Con**	**T1**	**T2**	**T3**	***p*-value**
Initial body weight (g)	1,670 ± 95	1,603 ± 106	1,586 ± 88	1,616 ± 100	0.94
Final body weight (g)	3,322 ± 368	3,302 ± 381	3,308 ± 393	3,274 ± 370	1.00
ADG (g/d)	34.4 ± 5.7	35.4 ± 5.8	35.9 ± 6.4	34.5 ± 5.7	0.97
ADFI (g/d)	148.1 ± 0.8	138.7 ± 1.7	143.4 ± 5.5	141.8 ± 4.4	0.38
F:G	4.3 ± 0.1^a^	3.9 ± 0.1^b^	4.0 ± 0.1^b^	4.1 ± 0.1^ab^	0.02

*ADG, average daily gain; ADFI, average daily feed intake; F:G, feed intake/body weight gain; T1, basal diet with 30 mg/kg of E. scaber supplementation; T2, basal diet with 80 mg/kg of E. scaber supplementation; T3, basal diet with 130 mg/kg of E. scaber supplementation*.

a,b *Means with different superscripts within a row differ (p < 0.05), n = 6*.

### Carcass Parameters

As shown in Table 3, the T3 group was higher (*p* < 0.05) than the Con group in the semi-eviscerated. The rates of carcass yield, eviscerated, breast muscle, leg muscle, abdominal fat, skin and subcutaneous fat, heart, liver, and gizzard were not affected (*p* > 0.05) by dietary supplementation with *E. scaber*.

**Table 3:** Effects of dietary supplementation with *Elephantopus scaber* on carcass parameters of ducks.

**Item, %**	**Con**	**T1**	**T2**	**T3**	***p*-value**
Carcass yield	86.0 ± 0.9	85.2 ± 0.9	84.7 ± 0.7	86.6 ± 0.8	0.43
Semi-eviscerated	79.1 ± 1.3^b^	79.9 ± 0.9^ab^	78.2 ± 1.0^b^	83.1 ± 1.6^a^	0.05
Eviscerated	67.8 ± 1.1	68.2 ± 0.9	66.9 ± 1.3	70.6 ± 1.3	0.19
Breast muscle	17.8 ± 0.4	17.1 ± 0.7	17.8 ± 0.9	17.0 ± 0.7	0.75
Leg muscle	10.3 ± 0.7	10.3 ± 0.9	10.6 ± 0.6	9.9 ± 1.0	0.64
Abdominal fat	2.7 ± 0.5	2.6 ± 0.4	3.3 ± 0.4	3.67 ± 0.7	0.41
Skin with subcutaneous fat	28.2 ± 1.5	28.3 ± 1.6	31.9 ± 1.7	28.3 ± 0.5	0.16
Heart	0.6 ± 0.02	0.6 ± 0.02	0.7 ± 0.03	0.8 ± 0.09	0.30
Liver	1.5 ± 0.09	1.5 ± 0.18	1.4 ± 0.08	1.5 ± 0.10	0.79
Gizzard	1.7 ± 0.08	1.9 ± 0.21	1.7 ± 0.08	1.8 ± 0.09	0.56

a,b *Means with different superscripts within a row differ (p < 0.05), n = 6*.

### Serum Biochemistry

**Figure 1:**
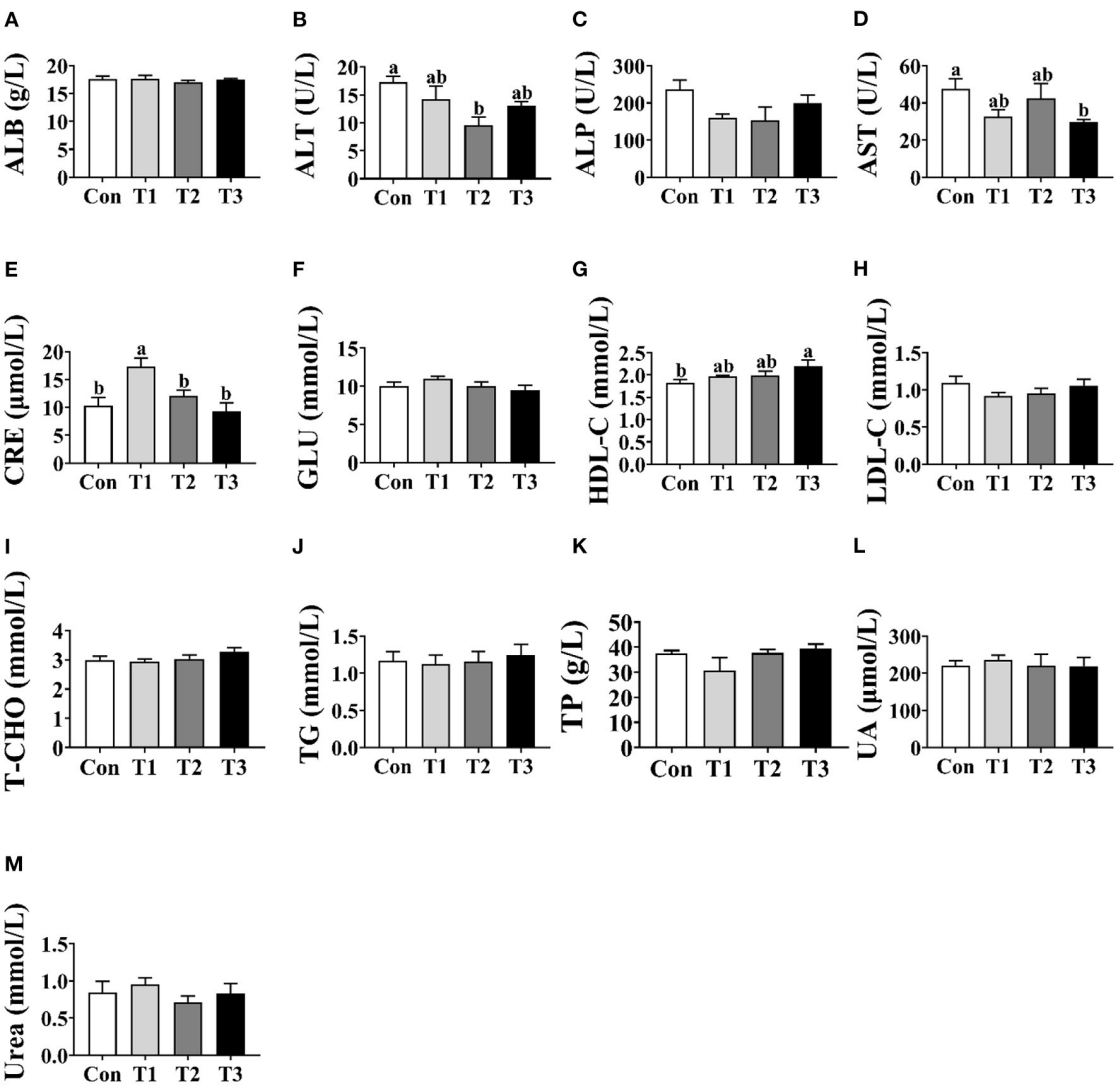
Effects of dietary supplementation with *Elephantopus scaber* on serum biochemical indexes of ducks. The concentrations of ALB **(A)**, ALT **(B)**, ALP **(C)**, AST **(D)**, CRE **(E)**, GLU **(F)**, HDL-C **(G)**, LDL-C **(H)**, T-CHO **(I)**, TG **(J)**, TP **(K)**, UA **(L)**, and urea **(M)** in serum. ALB, albumin; ALT, alanine aminotransferase; ALP, alkaline phosphatase; AST, aspartate aminotransferase; CRE, creatinine; GLU, glucose; HDL-C, high-density lipoprotein-cholesterol; LDL-C, low-density lipoprotein-cholesterol; T-CHO, total cholesterol; TG, triglyceride; TP, total protein; UA, uric acid.

As shown in Figure 1, no differences (*p* > 0.05) were observed in the concentrations of ALB, ALP, GLU, LDL-C, T-CHO, TG, TP, UA, or urea among the four groups (Figures 1A,C,F,H–M). Compared with the Con group, the T2 group was shown to decrease (*p* < 0.05) the concentration of ALT in the serum (Figure 1B). In addition, the T3 group was lower (*p* < 0.05) than the Con group in the concentration of AST (Figure 1D) and was higher (*p* < 0.05) than the Con group in the concentration of HDL-C (Figure 1G). The highest concentration of CRE was observed in the T1 group (*p* < 0.05; Figure 1E).

### Inosinic Acid Concentration in Breast Muscle

**Figure 2:**
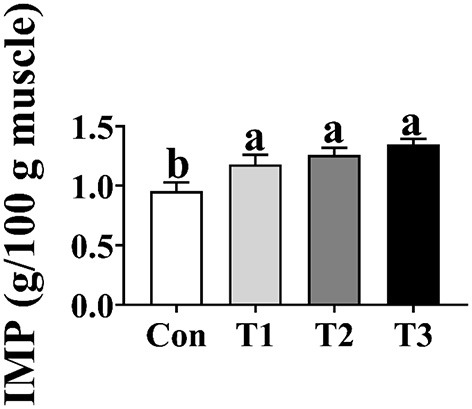
Effects of dietary supplementation with *Elephantopus scaber* on IMP content in breast muscle (g/100 g muscle). IMP, inosinic acid.

As shown in Figure 2, the concentration of IMP in the T1, T2, and T3 groups was higher (*p* < 0.05) than that in the Con group; however, there was no difference in the concentration of IMP in the breast muscle among the T1, T2, and T3 groups (*p* > 0.05).

### Fe, Se, and Zn Contents in Breast Muscle

As shown in Table 4, the Con group was higher than the T2 or T3 group in the Zn content. No difference (*p* > 0.05) was observed in the Fe or Se contents among the four groups.

**Table 4:** Effects of dietary supplementation with *Elephantopus scaber* on Fe, Se, and Zn contents in breast muscle (mg/kg muscle).

**Item**	**Con**	**T1**	**T2**	**T3**	***p*-value**
Fe	53.5 ± 3.3	49.5 ± 2.5	47.6 ± 1.8	47.1 ± 1.5	0.24
Se	0.4 ± 0.03	0.4 ± 0.01	0.4 ± 0.02	0.3 ± 0.03	0.15
Zn	16.5 ± 0.9^a^	14.4 ± 0.4^ab^	13.5 ± 0.7^b^	13.4 ± 0.6^b^	0.02

a,b *Means with different superscripts within a row differ (p < 0.05), n = 6*.

### Amino Acid Contents in Breast Muscle

As shown in Table 5, the contents of Phe, Ala, Gly, Glu, Arg, Lys, Tyr, Leu, Ser, Thr, Asp, and total AAs in the breast muscle were the highest (*p* < 0.05) in the T2 group. Compared with those in the Con group, the contents of Phe, Gly, Glu, Tyr, Leu, Ser, Thr, and Asp were increased (*p* < 0.05) in the T1 and T3 groups. The T3 group was higher than the Con group in the content of total AAs (*p* < 0.05).

**Table 5:** Effects of dietary supplementation with *Elephantopus scaber* on amino acid contents in breast muscle (g/100 g muscle).

**Item**	**Con**	**T1**	**T2**	**T3**	***p*-value**
Phe	0.90 ± 0.01^c^	0.93 ± 0.02^ab^	1.00 ± 0.01^a^	0.94 ± 0.01^ab^	0.03
Ala	1.43 ± 0.02^b^	1.45 ± 0.02^b^	1.53 ± 0.01^a^	1.47 ± 0.01^b^	0.01
Met	0.61 ± 0.01	0.62 ± 0.01	0.64 ± 0.01	0.63 ± 0.01	0.16
Pro	0.86 ± 0.01	0.89 ± 0.02	0.89 ± 0.01	0.93 ± 0.02	0.08
Gly	0.96 ± 0.01^c^	0.99 ± 0.01^b^	1.05 ± 0.01^a^	1.00 ± 0.01^b^	<0.01
Glu	3.20 ± 0.02^c^	3.32 ± 0.05^b^	3.48 ± 0.03^a^	3.36 ± 0.02^b^	<0.01
Arg	1.46 ± 0.02^b^	1.50 ± 0.02^b^	1.59 ± 0.02^a^	1.52 ± 0.01^b^	<0.01
Lys	1.96 ± 0.02^b^	2.01 ± 0.04^b^	2.13 ± 0.02^a^	2.04 ± 0.02^b^	<0.01
Tyr	0.78 ± 0.01^c^	0.81 ± 0.02^b^	0.85 ± 0.01^a^	0.81 ± 0.01^b^	<0.01
Leu	1.84 ± 0.02^b^	1.91 ± 0.04^a^	2.02 ± 0.03^a^	1.92 ± 0.02^a^	<0.01
Ser	0.87 ± 0.02^b^	0.93 ± 0.01^a^	0.94 ± 0.01^a^	0.92 ± 0.01^a^	<0.01
Thr	1.07 ± 0.01^c^	1.12 ± 0.01^b^	1.16 ± 0.01^a^	1.12 ± 0.01^b^	<0.01
Asp	2.03 ± 0.01^d^	2.09 ± 0.02^c^	2.22 ± 0.01^a^	2.16 ± 0.01^b^	<0.01
Val	1.13 ± 0.03	1.12 ± 0.05	1.20 ± 0.05	1.13 ± 0.05	0.55
Ile	0.99 ± 0.03	0.98 ± 0.06	1.06 ± 0.05	0.99 ± 0.05	0.62
His	0.63 ± 0.01	0.63 ± 0.01	0.68 ± 0.02	0.65 ± 0.01	0.08
Total AAs	20.73 ± 0.17^c^	21.32 ± 0.35^bc^	22.42 ± 0.24^a^	21.58 ± 0.20^b^	<0.01

*Total AAs, total amino acids*.

a−d *Means with different superscripts within a row differ (p < 0.05), n = 6*.

### Fatty Acid Contents in Breast Muscle

As shown in Table 6, dietary supplementation with *E. scaber* had no effects (*p* > 0.05) on the contents of C14:0, C16:0, C18:1*n*−9, C18:3*n*−6, C20:3*n*−6, C20:4, C24:0, C22:6ns, or monounsaturated fatty acid (MUFA) in the breast muscle. The content of C16:1 was highest in the Con group (*p* < 0.05). The T1 group was higher than the Con and T2 groups in the content of C18:0. The content of saturated fatty acid (SFA) was lower in the T2 group than that in the Con, T1, or T3 group, and that of polyunsaturated fatty acid (PUFA) was higher in the T2 group than in the Con group.

**Table 6:** Effects of dietary supplementation with *Elephantopus scaber* on fatty acid contents in breast muscle (%).

**Item**	**Con**	**T1**	**T2**	**T3**	***p*-Value**
C14:0	0.58 ± 0.02	0.65 ± 0.14	0.48 ± 0.04	0.52 ± 0.03	0.42
C16:0	24.30 ± 0.39	23.55 ± 0.26	20.62 ± 1.71	22.69 ± 0.73	0.06
C16:1	2.51 ± 0.09^a^	2.02 ± 0.07^b^	2.10 ± 0.16^b^	2.04 ± 0.08^b^	0.01
C18:0	9.82 ± 0.13^bc^	11.31 ± 0.49^a^	9.51 ± 0.50^c^	10.68 ± 0.30^ab^	0.02
C18:1*n*−9	34.29 ± 1.82	30.10 ± 1.77	29.77 ± 3.69	32.07 ± 2.00	0.49
C18:2*n*−6	18.11 ± 1.69^b^	20.08 ± 0.67^b^	27.28 ± 3.41^a^	19.45 ± 0.52^b^	0.01
C18:3*n*−6	0.91 ± 0.05	0.83 ± 0.05	0.77 ± 0.06	0.02 ± 0.007	0.27
C20:3*n*−6	0.35 ± 0.20	0.34 ± 0.09	0.45 ± 0.09	0.46 ± 0.03	0.42
C20:4	6.70 ± 0.47	8.06 ± 0.56	6.59 ± 0.58	7.77 ± 0.45	0.14
C24:0	1.88 ± 0.11	2.42 ± 0.15	1.87 ± 0.24	2.14 ± 0.18	0.14
C22:6ns	0.54 ± 0.04	0.63 ± 0.06	0.56 ± 0.07	0.63 ± 0.06	0.63
SFA	36.58 ± 0.28^a^	37.93 ± 0.81^a^	32.48 ± 2.08^b^	35.98 ± 1.01^a^	0.03
MUFA	36.80 ± 1.79	32.14 ± 1.83	31.87 ± 3.83	34.13 ± 2.00	0.46
PUFA	26.62 ± 2.05^b^	29.93 ± 1.16^ab^	35.65 ± 3.38^a^	29.89 ± 1.33^ab^	0.05

*SFA (saturated fatty acid) = C14:0 + C16:0 + C18:0 + C24:0*.

*MUFA (monounsaturated fatty acid) = C16:1 + C18:1n−9*.

*PUFA (polyunsaturated fatty acids) = C18:2n−6 + C18:3n−6 + C20:3n−6 + C20:4 + C22:6ns*.

a−c *Means with different superscripts within a row differ (p < 0.05), n = 6*.

### Intestinal Morphology

**Figure 3:**
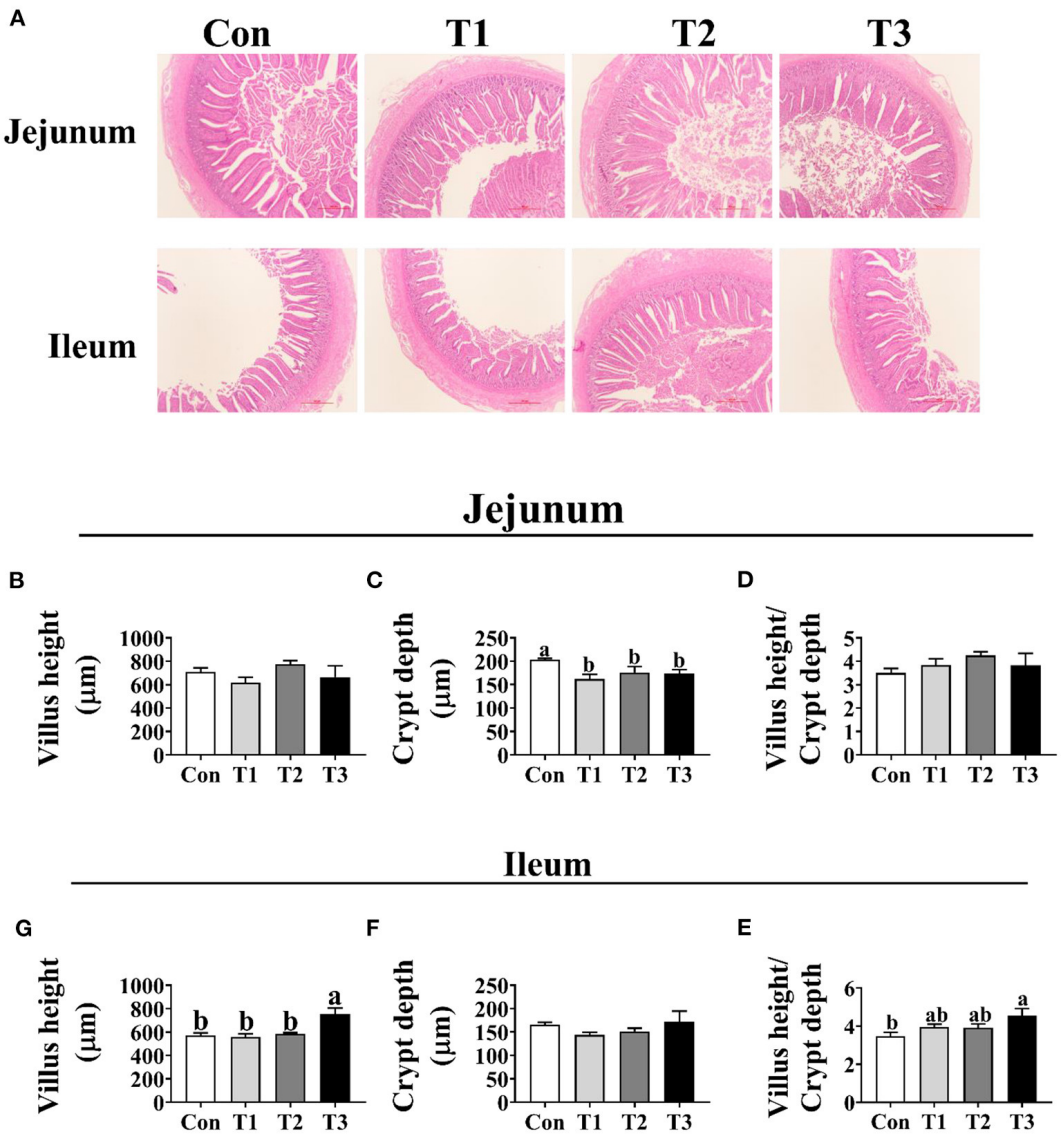
Effects of dietary supplementation with *Elephantopus scaber* on intestinal morphology of duck. **(A)** The images of the jejunum and ileum morphology; bar = 500 μm. Summarized date of villus height, crypt depth, and the ratio of villus height to crypt depth in jejunum **(B–D)** and ileum **(E–G)**.

As shown in Figure 3, the crypt depth of jejunum was significantly decreased in the T1, T2, and T3 groups than in the Con group (*p* < 0.05); however, no difference (*p* < 0.05) was observed in the jejunal villus height and the ratio of villus height to crypt depth among the four groups. In the ileum, the T3 group was higher (*p* < 0.05) than the Con group in the villus height and the ratio of villus height to crypt depth.

### Microbiota Community Composition

As shown in Table 7, dietary supplementation with *E. scaber* showed no effect on observed OTUs, Shannon, Simpson, or Chao1 index of microbiota. At the phylum level (Table 8), the most dominant phyla among microbiota communities (>1%) were Firmicutes, Bacteroidetes, Proteobacteria, Fusobacteria, and Actinobacteria; these microbiotas accounted for more than 93% of the total microbiota found in fecal samples. The abundances of Firmicutes and Bacteroidetes were the highest in the T2 group. The T3 group had a trend to increase the abundance of Fusobacteria. At the genus level (Table 9), the most dominant genus among microbiota communities were *Bacteroides, Fusobacterium, Ruminiclostridium_9, Desulfovibrio, Lachnospiraceae, Clostridiales_vadinBB60_group, Intestinimonas, Gorbachella, Oscillospira, Alistipes*, and *Erysipelatoclostridium*. Compared with the Con group, the T1 and T2 groups displayed a higher abundance of *Subdoligranulum* (*p* < 0.05). The abundances of *Fusobacterium* and *Ruminiclostridium_9* were the highest in T3, and the abundance of *Desulfovibrio* was the highest in the T1 group.

**Table 7:** Observed OTUs and alpha diversity of colonic bacterial community of ducks with different dietary treatments (*n* = 6).

**Item**	**Con**	**T1**	**T2**	**T3**	***p*-value**
Observed OTUs	758.50 ± 60.40	730.56 ± 58.77	681.78 ± 56.98	737.75 ± 93.85	0.94
Shannon	7.06 ± 0.25	7.15 ± 0.21	7.10 ± 0.24	7.28 ± 0.31	0.93
Simpson	0.97 ± 0.01	0.97 ± 0.01	0.97 ± 0.01	0.98 ± 0.01	0.81
Chao1	732.25 ± 84.13	761.64 ± 62.10	703.92 ± 59.51	763.80 ± 99.39	0.94

*OTUs, operational taxonomic units*.

**Table 8:** Phylum-level relative abundances of fecal microbiota in ducks with different dietary treatments (%).

Phylum^*^	**Con**	**T1**	**T2**	**T3**	***p*-value**
p_Firmicutes	39.63	41.41	44.51	42.64	0.61
p_Bacteroidetes	27.44	26.99	29.58	26.50	0.83
p_Proteobacteria	18.73	19.88	11.98	16.74	0.13
p_Fusobacteria	5.83	3.66	5.57	7.04	0.07
p_Actinobacteria	2.02	2.39	2.40	1.08	0.30
p_Synergistetes	0.99	1.26	0.73	1.65	0.38
p_Epsilonbacteraeota	0.54	0.30	0.55	0.19	0.54
p_Elusimicrobia	0.16	0.11	0.06	0.17	0.39
p_Tenericutes	0.22	0.11	0.12	0.26	0.61
p_Deferribacteres	0.51	0.43	0.18	0.51	0.85

* *Top 10 most abundant species are listed. The relative species abundances of gut microbial communities were analyzed using the Kruskal–Wallis test, n = 6*.

**Table 9:** Genus-level relative abundances of fecal microbiota in ducks with different dietary treatments (%).

Genus^*^	**Con**	**T1**	**T2**	**T3**	***p*-value**
*g_Bacteroides*	16.28	18.01	18.75	16.99	0.68
*g_Fusobacterium*	5.81	3.66	5.57	7.02	0.07
*g_Ruminiclostridium_9*	2.23	1.80	1.59	2.43	0.09
*g_Desulfovibrio*	15.16	18.58	10.33	13.50	0.11
*g_Lachnospiraceae*	3.71	5.85	8.37	2.79	0.27
*g_Clostridiales_vadinBB60_group*	2.98	2.16	1.58	2.73	0.41
*g_Intestinimonas*	2.99	3.04	2.92	3.32	0.92
*g_Gorbachella*	1.31	1.12	0.69	1.58	0.12
*g_Bacteroidales_unclassified*	1.00	1.77	1.99	0.31	0.16
*g_Faecalibacterium*	0.61	0.92	1.29	1.27	0.44
*g_Ruminococcus_2*	1.65	1.35	1.51	0.95	0.98
*g_Alistipes*	2.65	2.27	3.08	2.64	0.97
*g_Olsenella*	1.04	1.25	0.94	0.76	0.62
*g_Subdoligranulum*	0.97^b^	1.86^a^	1.50^a^	0.36^b^	0.03
*g_Oscillospira*	1.65^a^	1.15^ab^	1.03^b^	1.91^a^	0.05
*g_Erysipelatoclostridium*	1.17	1.07	1.89	0.68	0.24

* *Genus with proportion under 1.00% are not listed. The relative species abundances of gut microbial communities were analyzed using the Kruskal–Wallis test, n = 6*.

a,b *Means with different superscripts within a row differ (p < 0.05)*.

### Correlation Analysis of Environmental Factors

**Figure 4:**
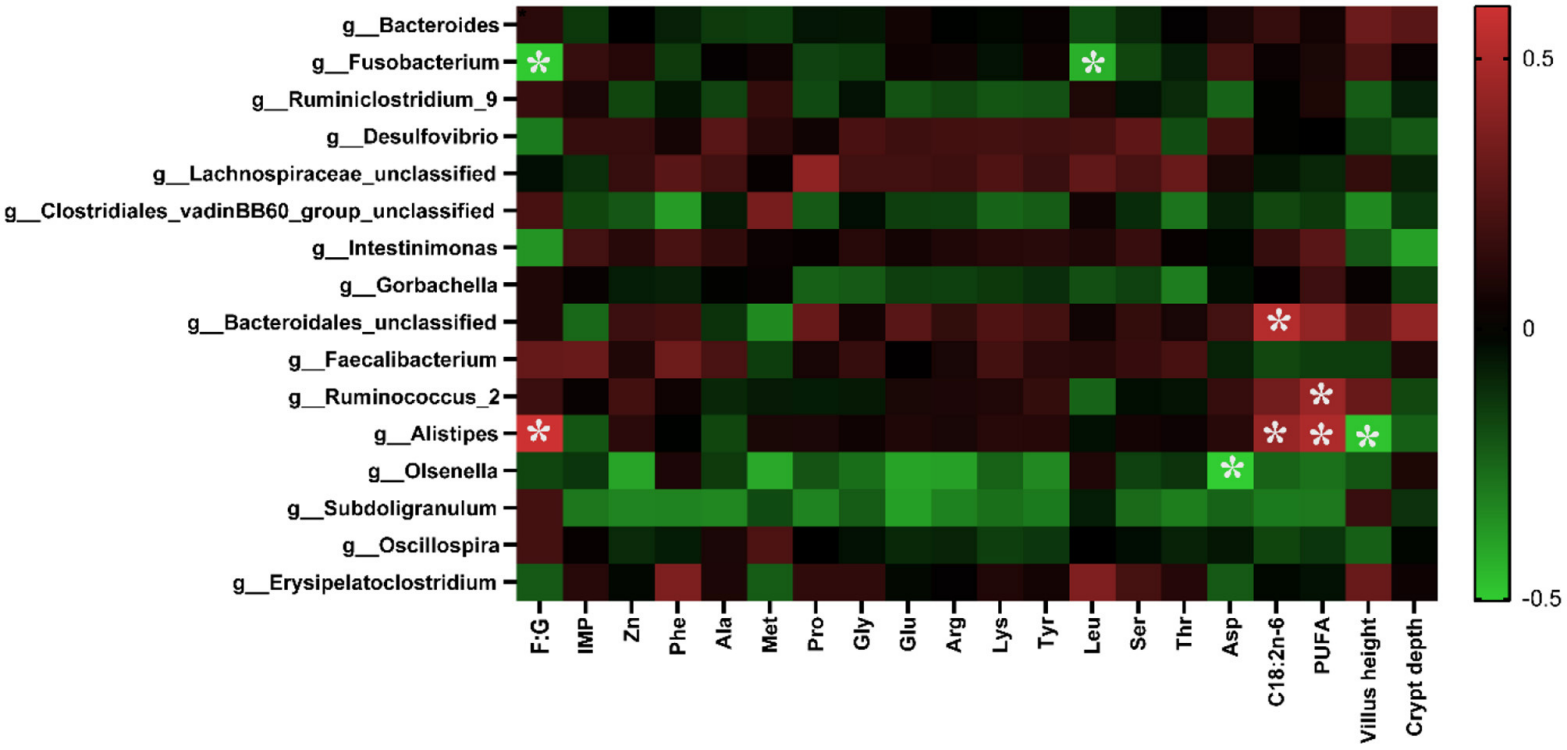
Correlation analysis of environmental factors. The colors range from green (negative correlation) to red (positive correlation). The data were analyzed using Pearson's correlation coefficient. *In green grid indicates a negative correlation (*p* < 0.05), whereas in the red grid represents a positive correlation (*p* < 0.05).

As shown in Figure 4, the abundance of *Alistipes* was positively correlated with the F:G, the contents of C18:2*n*−6, and PUFA in the breast muscle (*p* < 0.05), while it was negatively correlated with the villus height (*p* < 0.05). The abundance of *Olsenella* was negatively correlated with the content of Asp in the muscle (*p* < 0.05). The abundance of *Ruminococcus_2* was positively correlated with the level of PUFA in the muscle (*p* < 0.05), and that of *Fusobacterium* was negatively correlated with the F:G and the content of Leu in muscle. In addition, the abundance of Bacteroidales was positively correlated with the content of C18:2*n*−6 in the breast muscle.

## Discussion

Studies have reported that herbs can improve animal growth performances and meat quality (15, 16). *E. scaber* is a traditional herbal medicine and plays an important role in animal health such as in antimicrobial, antioxidant, anti-inflammatory, and wound healing activities (17). However, the effects of dietary supplementation with *E. scaber* on the growth performance, meat quality, intestinal development, and microbiota composition of ducks are not elucidated. In the present study, we found that dietary supplementation with *E. scaber* had no effects on final body weight, ADFI, and ADG. However, a previous study reported that dietary supplementation with *E. scaber* extract significantly promoted final body weight, weight gain, and growth rate of fish (18). The discrepancy might be explained by difference in the animal model. Interestingly, a decrease in the F:G was observed in the T1 group, indicating that dietary supplementation with 30 mg/kg of *E. scaber* increased the feed conversion of ducks. In line with our results, a previous study reported that dietary inclusion of 5 g/kg of *E. scaber* extract significantly improved feed conversion ratio in Nile tilapia (18). To further investigate the beneficial role of *E. scaber* on duck's health, we evaluated the blood chemical parameter. In the present study, we found that the levels of ALT and AST in the serum were significantly lower in the T2 and T3 groups, respectively, which was consistent with a previous study (19). ALT and AST are indexes that reflect the liver injury of animal, and their levels in the serum were significantly increased in liver injury mouse (20), which indicates that dietary supplementation with *E. scaber* might alleviate the liver injury in ducks.

Meat provides essential nutrients including amino acids and fatty acids for humans. Amino acids not only are the building blocks of protein but also have key roles in the aroma and flavor profiles of meat. For example, Arg, Leu, and Phe present a bitter taste; Glu presents a fresh taste; and Gly, Ala, and Ser present a sweet taste (21). Therefore, improving meat nutritional composition has a vital role in producing high-quality duck meat. Here, we observed that dietary supplementation with 30 mg/kg of *E. scaber* increased the contents of Phe, Gly, Glu, Tyr, Leu, Ser, Thr, and Asp in the breast muscle, and that with 80 mg/kg of *E. scaber* increased the contents of Phe, Ala, Gly, Glu, Arg, Lys, Tyr, Leu, Ser, Thr, and total AAs in the breast muscle. Another important finding is that dietary supplementation with 80 mg/kg of *E. scaber* increased the contents of C18:2*n*−6 (linoleic acid) and PUFA in the breast muscle. Linoleic acid is an essential fatty acid, and consumption of PUFA is beneficial to human health in humans (22, 23). These results suggest that dietary supplementation with 80 mg/kg of *E. scaber* increased meat quality of ducks though modulating amino acid and fatty acid composition. Increased amino acids and fatty acids in the breast muscle suggested that dietary supplementation with *E. scaber* increased the flavor profiles of meat. To support this result, we determined the IMP content in the breast muscle. In the present study, we found that dietary supplementation with *E. scaber* increased IMP content in the breast muscle. IMP is one of the major contributors to flavor in meat (24). This result further confirmed that dietary supplementation with *E. scaber* increased the meat quality of ducks.

The morphology of intestine is a key factor influencing nutrient absorption (25). Given the increased feed conversion in ducks fed with *E. scaber*, we suspected that dietary supplementation with *E. scaber* might improve the intestine development. Therefore, the intestine morphology of ducks was investigated. In the present study, we found that dietary supplementation with 30 or 80 mg/kg decreased the crypt depth in jejunum. In addition, dietary supplementation with 130 mg/kg of *E. scaber* increased the villus height in ileum. In support of our results, a previous study showed that *E. ulmoides* leaf extract treatment increased villus height of the duodenum and jejunum in weaned piglets (26). These results suggest that dietary supplementation with *E. scaber* increased intestinal development.

Intestinal microbiota plays an important role in host physiology (27). Changes in diets can modulate the composition of the intestinal microbiota, which in turn affects animal physiology and metabolism function. Due to the changed meat quality and intestine morphology, we investigated the effects of dietary supplementation with *E. scaber* on colonic microbiota composition. In the present study, no difference was observed in the Shannon, Simpson, or Chao 1 index, indicating that dietary *E. scaber* supplementation showed no effects on microbial diversity. In the present study, Bacteroidetes and Firmicutes were the dominant phyla in feces, which was consistent with a previous study (28). In addition, the T3 group had a trend to increase the abundance of Fusobacteria in feces. *Fusobacterium nucleatum* influences stages of colorectal cancer progression and causes opportunistic infections (29), which suggests that dietary supplementation with 130 mg/kg of *E. scaber* might exert a negative impact on ducks' health. At the genus level, the abundance of *Subdoligranulum* was higher in the T2 group than in the Con group. *Subdoligranulum* is a butyrate-producing bacterium (30). Evidence showed that microbial-derived butyrate promotes intestinal epithelial barrier function through IL-10 receptor-dependent repression of Claudin-2 (31). In addition, butyrate-producing bacteria supplemented in diets increased butyrate production and enhanced intestinal epithelial barrier integrity (32). Therefore, we suspected that dietary supplementation with 80 mg/kg of *E. scaber* decreased the crypt depth in jejunum through modulating the abundance of *Subdoligranulum*. However, the mechanism needs further investigation. To investigate the relationship between environmental factors, a Pearson's correlation coefficient was conducted. In the present study, we observed that the abundance of *Alistipes* was negatively correlated with villus height and was positively correlated with F:G. *Alistipes* was suggested as a harmful bacterium and was positively correlated with inflammation (33), indicating *Alistipes* might act as a target bacteria for modulating intestinal development and nutrient utilization.

## Conclusions

Dietary supplementation with 30 mg/kg of *E. scaber* increased the feed conversion of ducks, and that with 80 mg/kg of *E. scaber* increased meat quality through improving amino acids, IMP, and fatty acid profile in the breast muscle. The results of the present study contribute to a better understanding of the role of *E. scaber* as a feed additive in duck production and provide basic knowledge for producing high-quality duck meat for human consumption based on nutritional regulation. Future work can investigate and identify the effective compounds in *E. scaber* involved in improvement of duck production.

## Data Availability Statement

The datasets presented in this study can be found in online repositories. The names of the repository/repositories and accession number(s) can be found below: NCBI (accession: PRJNA750146).

## Ethics Statement

The animal study was reviewed and approved by Care and Use of Chinese Academy of Tropical Agricultural Sciences.

## Author Contributions

CH, LG, and TX contributed to the study design, conducted the animal experiments, and wrote the manuscript. CH, ML, FJ, WS, DW, and WP executed the lab analysis. DL and QL performed the statistical analysis. HD and HZ revised the paper. All authors carefully read and approved the final revision of the manuscript.

## Funding

This present work was jointly supported by China Agriculture Research System of MOF and MARA, Central Public-interest Scientific Institution Basal Research Fund for Chinese Academy of Tropical Agricultural Sciences (1630032017034 and 1630032021004) and National Natural Science Foundation of China (31972553).

## Conflict of Interest

The authors declare that the research was conducted in the absence of any commercial or financial relationships that could be construed as a potential conflict of interest.

## Publisher's Note

All claims expressed in this article are solely those of the authors and do not necessarily represent those of their affiliated organizations, or those of the publisher, the editors and the reviewers. Any product that may be evaluated in this article, or claim that may be made by its manufacturer, is not guaranteed or endorsed by the publisher.
